# Strong spatial genetic structure in five tropical *Piper* species: should the Baker–Fedorov hypothesis be revived for tropical shrubs?

**DOI:** 10.1002/ece3.40

**Published:** 2011-12

**Authors:** E Lasso, J W Dalling, E Bermingham

**Affiliations:** 1 Smithsonian Tropical Research InstituteApartado Postal 0843-03092, Panamá, República de Panamá; 2 Departamento de Ciencias Biológicas, Universidad de los AndesCarrera 1E No 18A-10, AA 4976, Bogotá, Colombia; 3 University of Illinois, Plant Biology149 Morrill MC-116, Urbana, IL 61801

**Keywords:** AFLP, Barro Colorado Island, Clonal reproduction, Gene flow, Piperaceae

## Abstract

Fifty years ago, Baker and Fedorov proposed that the high species diversity of tropical forests could arise from the combined effects of inbreeding and genetic drift leading to population differentiation and eventually to sympatric speciation. Decades of research, however have failed to support the Baker–Fedorov hypothesis (BFH), and it has now been discarded in favor of a paradigm where most trees are self-incompatible or strongly outcrossing, and where long-distance pollen dispersal prevents population drift. Here, we propose that several hyper-diverse genera of tropical herbs and shrubs, including *Piper* (>1,000 species), may provide an exception. Species in this genus often have aggregated, high-density populations with self-compatible breeding systems; characteristics which the BFH would predict lead to high local genetic differentiation. We test this prediction for five *Piper* species on Barro Colorado Island, Panama, using Amplified Fragment Length Polymorphism (AFLP) markers. All species showed strong genetic structure at both fine- and large-spatial scales. Over short distances (200–750 m) populations showed significant genetic differentiation (*Fst* 0.11–0.46, *P* < 0.05), with values of spatial genetic structure that exceed those reported for other tropical tree species (*Sp* = 0.03–0.136). This genetic structure probably results from the combined effects of limited seed and pollen dispersal, clonal spread, and selfing. These processes are likely to have facilitated the diversification of populations in response to local natural selection or genetic drift and may explain the remarkable diversity of this rich genus.

## Introduction

Evolutionary biologists have long sought an explanation for the high diversity of closely related species that coexist in tropical forests. One of the first micro-evolutionary explanations for the origin of this diversity, the Baker–Fedorov hypothesis (BFH), was based on the expectation of restricted gene flow in tropical trees. Baker (1959) and Fedorov (1966) proposed that tropical trees should be highly self-fertilized or inbred, due to their low population density. Thus, genetic drift should promote incipient speciation over relatively limited spatial scales. As a result, many closely related species could occur sympatrically. However, after several decades of research this hypothesis has been discarded in light of the collective evidence indicating that many tropical tree species are self-incompatible (Bawa 1979; Bawa et al. 1985) and have high rates of out-crossing and long-distance pollen dispersal (reviewed by Ward et al. 2005). Reported distances of seed dispersal mediated by animals are generally in the range of 100 m to 1 km (e.g., Murray 1988; Westcott and Graham 2000; Russo et al. 2006) and of pollen movement in the range of 100 m to 14 km (e.g., Hamrick and Murawski 1990; Stacey et al. 1996; Nason et al. 1998; Dick et al. 2008). The majority of these estimates, however, are for canopy trees.

Trees differ from shrubs and herbs in several respects. First, trees tend to occur at lower population densities (Hubbell and Foster 1983), which requires pollinators to move large distances between individuals (Stacey et al. 1996). Second, trees have more resources available to invest in fruit crops, which contributes to making foraging on these species energetically viable for large-bodied dispersers with larger home ranges, and therefore increases dispersal distances (Carbone et al. 1999). Third, trees tend to be mostly out-crossed (Bawa 1979; Jaimes and Ramirez 1999; Ward et al. 2005; Devy and Davidar 2006), whereas shrubs and herbs often have self-compatible and mixed-mating breeding systems (Bawa 1990, 1992). It is possible, then, that gene flow is more extensive in trees than it is in understory shrubs and herbs, which also often occur in aggregated, dense populations.

The majority of floristic diversity in tropical forests is found in the understory as shrubs or small trees. For example, on Barro Colorado, Panama, almost 63% of 320 species on the 50 ha forest dynamic plot are understory trees, midstory trees or shrubs, compared to 35% that are canopy trees (Hubbell et al. 2005). Some of the most diverse tropical woody plant genera such as *Piper* (>1,000 species), *Psychotria* (∼2,000 species), *Miconia* (>1,000 species), and *Inga* (>300 species) are composed mostly of shrubs and small trees that often grow sympatrically (Gentry and Dodson 1987) and that have diversified recently (Richardson et al. 2001). Many species within these genera also show some capacity to spread asexually (Kinsman 1990; Greig 1993; Sagers 1993; Lasso et al. 2009), which could favor the establishment and subsequent survival of isolated populations. Genetic variation can arise in those isolated populations by somatic mutation (Lasso et al. 2011), but whether these populations can diversify and evolve independently in response to local natural selection or genetic drift (Slatkin 1985; Ellstrand 1992) will depend in part on how restricted gene flow is among their populations.

Despite shrubs spectacular diversity, and their obvious differences from canopy trees, there remain few studies of shrub genetic structure and gene flow. Here, we investigated the genetic structure of five shrub species in the genus *Piper*, one of the most speciose genera in Neotropical forests (>1,000 species; Gentry 1990) to evaluate one of the predictions of the BFH about the spatial scale of gene flow. We hope that this initial dataset will stimulate more research on this important, yet overlooked component of tropical forest. We focus on five species with different life history characteristics; three commonly found in the understory (*Piper darienensis* C. DC, *Piper cordulatum* C. DC., and *Piper aequale* Vahl.), and two in clearings (*Piper dilatatum* L. C. Rich, and *Piper marginatum* Jacq). *Piper* species share pollinators (Semple 1974; Fleming 1985; Figueiredo and Sazima 2000) and seed dispersers (Thies and Kalko 2004), but differ in their degree of clonality (Greig 1993; Lasso et al. 2009) and in their mating systems (Figueiredo and Sazima 2000; E. Lasso, unpublished data). We expected to observe higher genetic structure in the two understory species with higher levels of clonality than in clearing species and in one of the understory species with little clonal spread (see Table 1 for classification). We also expected to observe less genetic structure in *P. marginatum*, the only self-incompatible species studied (Hamrick and Godt 1996). To compare the degree of spatial genetic structure among species in our study, as well as to published studies of other shrub and tree species, we used the *Sp* statistic (Vekemans and Hardy 2004), which allows for quantitative comparisons among different studies. Additionally, we evaluated population differentiation using *Fst* and Bayesian analysis.

**Table 1:** Life history characteristics for five *Piper* shrub species. All species are insect-pollinated

Species	Habitat	Breeding system	Yearly seed production^(1)^	% asex. recruitment^(1)^	Seed disperser^(2)^	Flowering phenology^(2)^
*P. darienensis*	U	SC	177	42	Bt	Br
*P. cordulatum*	U	SC	940	36	Bt	Br
*P. aequale*	U	SC	8,606	7	Bt	Br
*P. marginatum*	G	SI	14,512	13	Bt, Bd, A	Co
*P. dilatatum*	G	SC	14,432	6	Bt, Bd, A	Co

Habitat: U = Understory; G = Gap.

Breeding system: SC = self-compatible; SI = self –incompatible.

Seed disperser: A = ants; Bt = Bats; Bd = birds.

Flowering and Fruiting: Br = brief; Co = continuous.

(1) = Lasso et al. 2009; (2) = Thies 1998.

## Materials and Methods

### Study site and study species

The study was conducted in tropical semi-deciduous forest on Barro Colorado Island (BCI), Panama (9°10′N, 79°51′W) that is described in detail by Leigh (1999). Annual rainfall on BCI averages 2,600 mm, with a pronounced dry season between January and April.

The genus *Piper* is an important component of tropical forests worldwide and is represented by 22 species on BCI (Croat 1978). Here, we focus on five species that differ in their life history characteristics as described in Table 1.

### Sampling scheme

To include both species variation in life history characteristics, and to assess population genetic differentiation, we analyzed five *Piper* species but limited our sampling to two populations per species. Populations of shade-tolerant species were sampled in two one-hectare plots 754 m apart in the forest understory. All plants present in the plot, growing at least 10 cm apart, were tagged, mapped, and genotyped. In plot 1, we collected young leaves from 182 ramets of *P. darienensis*, 166 of *P. aequale*, and 72 of *P. cordulatum*. In plot 2, we collected leaves from 167 ramets of *P. darienensis*, 59 of *P. aequale*, and 60 of *P. cordulatum*. Populations of the light-demanding species were sampled across two 35 × 35 m plots (plot 3 and plot 4) located 200 m apart in the laboratory clearing. In plot 3, we collected leaves from 33 plants of *P. dilatatum* and 43 of *P. marginatum*. In plot 4, we collected leaves from 43 plants of *P. dilatatum* and 41 of *P. marginatum*. The location of each plant in each plot is available in Lasso et al. (2011). The size of the laboratory clearing areas at the site did not permit the establishment of larger plots.

### DNA isolation, AFLP procedure, and clone identification

Leaves were collected and kept on ice until they were processed in the laboratory. On the day of collection, leaves were surface cleaned with 95% alcohol and left to dry in silica gel for 1 week. Twenty milligrams of dry tissue was ground using the FastPrep FP120 (MP Biomedicals, Irvine, CA, USA). DNA was extracted using a DNeasy 96 plant extraction kit (Qiagen Inc., Valencia, CA, USA) following the manufacturer's protocol. DNA concentrations were established by running DNA samples with Low DNA Mass™ Ladder (Invitrogen, Carlsbad, CA, USA) of known concentration on agarose gels.

AFLP analysis followed the method of Vos et al. (1995), but restriction digestion and ligation were performed separately. The PCR conditions, primer pair selection, genotyping procedures, and number of polymorphic loci for these species are described in detail in Lasso (2008). Genotype data were obtained by running the amplified samples in an ABI Prism 3130*xl* capillary electrophoresis machine, and the presence or absence of fragments was scored using Genescan and Genotyper software (version 3.7, Applied Biosystems, **Carlsbad, California, USA**). Clones and genetically distinct individuals were identified using the *Genotype* software (Meirmans and Van Tienderen 2004) as explained in Lasso (2008). In short, we estimated pairwise genetic distances among replicate samples of known clones and nonclones for each species to identify the amount of intraclonal genetic variation and set a threshold to classify samples as members of a clonal group or as unique genotypes. The threshold indicates the maximum dissimilarity that is allowed between individuals to still be considered clonemates and varied depending on the species, ranging from 0% to 5%.

### Fine scale spatial genetic structure

To characterize the fine-scale spatial genetic structure of mapped and genotyped individuals within plots, we used the program spag*e*d*i* version 1.2 (Hardy and Vekemans 2002). We estimated the kinship coefficient (*Fij*) for dominant markers developed by Hardy (2003). To compute this kinship coefficient, an estimate of the inbreeding coefficient must be included. Based upon the result of hand-pollination experiments (E. Lasso unpublished data), we used an inbreeding coefficient of F_I_ = 0 for *P. marginatum*, which we found to be self-incompatible, and an inbreeding coefficient of F_I_ = 0.5 for all remaining species because they had a mixed breeding system (E. Lasso, unpublished data). We also tested other inbreeding coefficients and found only a slight effect on patterns of spatial genetic structure (SGS). Given that this estimate has been proven to be fairly robust to moderate errors made on the assumed inbreeding coefficient (Hardy 2003) and that our results across different breeding scenarios were similar, we present here only the results obtained using these two inbreeding coefficients.

To test for SGS, we computed the average multilocus kinship coefficients per distance interval for the following distance classes: 2, 5, 10, 20, 30, 40, 50, 60, 70, 80, 90, and 100 m (for understory species), and 2, 5, 10, 20, and 40 m (for light-demanding species). To test the hypothesis that there was significant SGS, the observed regression slope of *F_ij_* on the natural logarithm of the physical distance between samples *i* and *j* (ln(*r_ij_*)), was compared with those obtained after 1,000 random permutations of individuals among positions. This procedure has the advantage that all the information is contained in one single-test statistic, and the results are independent of arbitrarily set distance intervals (Vekemans and Hardy 2004).

Additionally, we quantified the ‘*Sp*’ statistic as *Sp* = –*b*/(1 – *F*_1_), where *b* is the slope of the regression of *F_ij_* on ln(distance), and *F*_1_ is the mean *F_ij_* between individuals in the first distance class (here, 0–2 m). The *Sp* statistic is a synthetic way of calculating the strength of spatial genetic structure and enables simple comparisons to be made among species and to other published studies because it is less sensitive to sampling schemes than other measures of spatial genetic structure intensity (Vekemans and Hardy 2004). To determine how clonality affects fine-scale spatial genetic structure, two separate analyses were performed for each population, one including all ramets, and one for only genets or unique genotypes (excluding clones), selecting randomly from the database one ramet per clone to leave in the analysis. Comparisons between these two analyses were used to determine the relative contribution of clones to spatial genetic structure.

To assess whether shrub genetic structure might differ from that of canopy trees, we compared our *Sp* values obtained from five *Piper* species with those reported for 45 canopy tree species and another six shrubs reported in the following articles: Vekemans and Hardy (2004); Hardy et al. (2005); Jones and Hubbell (2006); Cloutier et al. (2007); Dick et al. (2008); Bizoux et al. (2009).

### Measuring population structure and gene flow

When using dominant markers, such as AFLP, it is recommended that several approaches be used to estimate population differentiation, thus providing greater confidence in data interpretation (Bonin et al. 2007). Here we used two approaches to assess how genetically differentiated *Piper* populations are (i.e., how extensive gene flow is). First, we calculated Wright's *Fst* statistic from estimates of allelic frequencies under different inbreeding scenarios (Zhivotovsky 1999). Second, we used a model-based method in conjunction with a Bayesian statistical approach to determine the number of subpopulations in our dataset and to assign individuals (probabilistically) to populations (Falush et al. 2007). By identifying putative migrants, we could infer patterns on the quantity and directionality of the gene flow for each species.

### Fst statistic approach

Wright's *Fst* statistic was calculated using the program AFLP-surv V.1.0 (Vekemans et al. 2002). To calculate *Fst*, the program first computes estimates of allelic frequencies at AFLP loci following the Bayesian method with a nonuniform prior distribution of allele frequencies. This has been shown to be superior to other methods used to estimate allele frequencies for dominant markers (Zhivotovsky 1999). As most of these species are self-compatible and can reproduce asexually, it is unlikely that their genotype frequencies are at Hardy–Weinberg equilibrium. In the absence of information on the exact level of inbreeding for the populations under study, we used three hypothesized values of the inbreeding coefficient (*F_is_* = 0, *F_is_* = 0.5, *F_is_* = 1.0) to observe the range of possible outcomes for each species. An inbreeding coefficient of *F*_is_ = 0 is typical for random mating, and, a coefficient of *F*_is_ = 1 indicates complete selfing. The significance of the genetic differentiation among populations was tested by comparison of the observed *Fst* with a distribution of *Fst* under the hypothesis of no genetic structure, obtained by means of 10,000 random permutations of individuals among populations. We also calculated Ф_pt_, a measure of population genetic differentiation that is analogous to *Fst* and is used specifically for AFLP and other binary data, using GenAlEx 6 (Peakall and Smouse 2006). The significance of departures from the null expectation of population genetic differentiation was tested by comparing the observed Ф_pt_ with the frequency distribution of permuted Ф_pt_ from 10,000 random permutations of individuals among populations.

### Bayesian approach using structure

To further explore population structure, and to identify migrants and admixed individuals, we used a Bayesian approach (Pritchard et al. 2000) implemented in the software *Structure* (ver. 2.2). This method uses a Markov chain Monte Carlo (MCMC) algorithm to account appropriately for the genotypic ambiguity inherent in dominant markers like AFLP (Falush et al. 2007). First, we chose the number of populations (*K*) that are most appropriate for interpreting the data (see below). Then, we examined the clustering of individuals in the *K* populations to identify the admixed individuals. On each run, the program places individuals into *K* panmictic groups, by minimizing deviations from Hardy–Weinberg equilibrium and linkage equilibrium. The program calculates an estimate of the posterior probability of the data for a given *K*, Pr(*X*|*K*) (Pritchard et al. 2000).

To choose the appropriate *K* and obtain insight into how the genetic variation was organized based on the clustering of individuals, we ran the program *Structure* (ver. 2.2) without prior information on the population of origin using values from *K* = 1 to *K* = 5. We generated a series of 10 independent runs for each value of *K* and we used the *ad hoc* statistic Δ*K* (Evanno et al. 2005) to select the best *K*. For all the runs, we assumed an admixture model with correlated allele frequencies and used a length of the burn-in of 100,000 and MCMC iterations of 200,000 each. We found that *K* = 2 is the most appropriate model for interpreting the data for all species.

To identify immigrant individuals in both populations, we ran the program using the prior information on the geographic sampling origin of the individuals, and leaving all the parameters as described above. In this analysis, two additional parameters had to be set: GENSBACK and MIGPRIOR. They correspond to the *G* and the *v* of Pritchard et al. (2000). *G* indicates the number of generations being tested and *v* represents the probability that an individual is an immigrant to any of the sampled populations. We first tested values of *v* between 0.001 and 0.1 and found consistent results indicating that the amount of information in the data was sufficient (Pritchard et al. 2000). Data presented are those obtained when using MIGPRIOR = 0.05. We set GENSBACK to 3 to calculate probability that each individual has a given amount of ancestry from the alternative populations coming from any of the past three generations.

All *Structure* analyses were done for the whole sample and for only genets (excluding clones). Because the results were similar, we present only the results from the analysis of the entire dataset.

## Results

### Fine scale spatial genetic structure within plots

**Figure 1:**
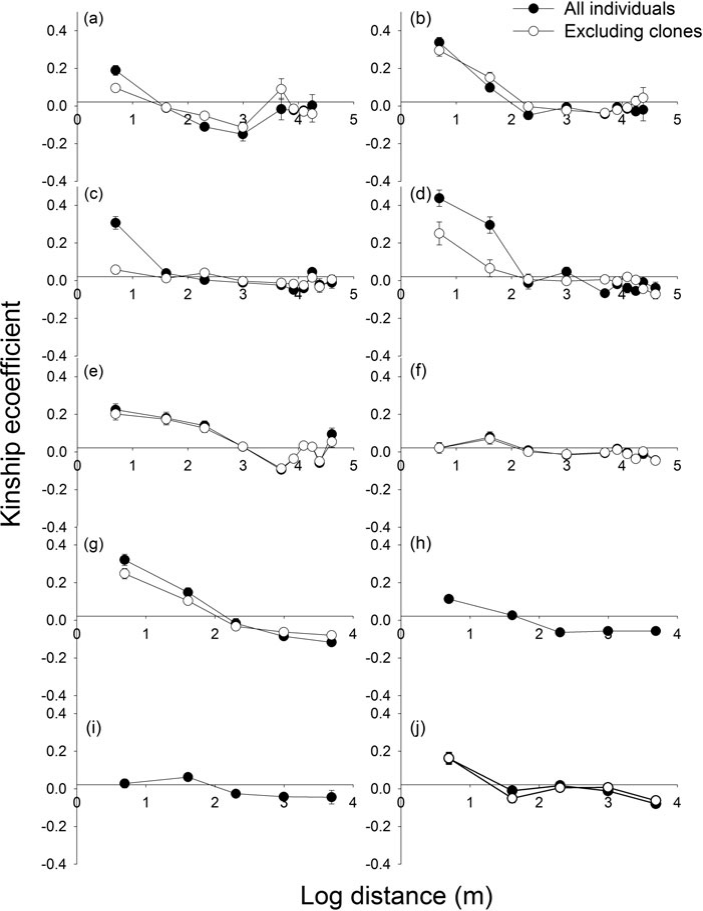
Correlograms of kinship coefficient (*Fij*) for all indiviuals in the population (clones and not clones) and for only genetically distinct shoots (excluding clones). Figures (A) and (B) are for *P. darienensis* in plot 1 and 2, figures (C) and (D) are for *P. cordulatum* in plot 1 and 2, figures (E) and (F) are for *P. aequale* plot 1 and 2, figures (G) and (H) are for *P. marginatum* plot 3 and 4; and figures (I) and (J) are for *P. dilatatum* plot 3 and 4.

The spatial autocorrelation analysis revealed strong and significant SGS in all species and all sites. The slope of the regression line (b) between the kinship coefficient and the natural logarithm of the distance between individuals was highly significant in all instances, even after removing clones from the analysis (Table 2). However, the degree of SGS, varied among sites and species (Table 2; Fig. 1).

**Table 2:** The slope *b* of the regression of pairwise kinship coefficients on the logarithm of geographic distance, the kinship coefficient between neighbors plants (F_(1)_), the *Sp* statistic estimated, and the significance level of the test (*P*) for the regression slope

Species			n	*b*	F_(1)_	*Sp*	*P*
*P. darienensis*	Plot 1	All	182	–0.0820	0.1900	0.1012	0
		Excluding clones	105	–0.0390	0.0951	0.0431	0
	Plot 2	All	164	–0.0766	0.3283	0.1141	0
		Excluding clones	98	–0.0705	0.2947	0.1000	0
*P. cordulatum*	Plot 1	All	72	–0.0518	0.3070	0.0747	0
		Excluding clones	46	–0.0390	0.0951	0.0431	0
	Plot 2	All	60	–0.0856	0.4377	0.1522	0
		Excluding clones	38	–0.0372	0.2508	0.0496	0
*P. aequale*	Plot 1	All	166	–0.1062	0.2241	0.0479	0
		Excluding clones	156	–0.0140	0.0581	0.0149	0
	Plot 2	All	59	–0.0164	0.0215	0.0168	0.007
		Excluding clones	55	–0.0150	0.0209	0.0153	0.009
*P. dilatatum*	Plot 3	All	26	–0.0318	0.0286	0.0328	0.007
		There were no clones in this population					
	Plot 4	All	43	–0.0484	0.1619	0.0578	0
		Excluding clones	39	–0.0372	0.1627	0.0444	0.009
*P. marginatum*	Plot 3	All	43	–0.1297	0.3207	0.1909	0
		Excluding clones	32	–0.0982	0.2478	0.1305	0
	Plot 4	All	41	–0.0703	0.1126	0.0792	0
		There were no clones in this population					

Results are presented for each of the two 1-ha plots studied and for all individuals in the population (All) and for only genets (Excluding clones).

For most species, plants located within 2 m were closely related. Kinship values were particularly high at the first distance class, with F_(1)_ ranging between 0.02 and 0.43. Values of 0.25 and 0.125 are expected for full and half-siblings, respectively; therefore some clonal spread seems to contribute to population growth. However, some F_(1)_ values remained above 0.25 even when ramets are excluded, suggesting that some biparental inbreeding is taking place. While clonality is not the only factor responsible for the strong SGS observed, slopes were two or three times higher when clones were included in the analysis (Table 2).

**Figure 2:**
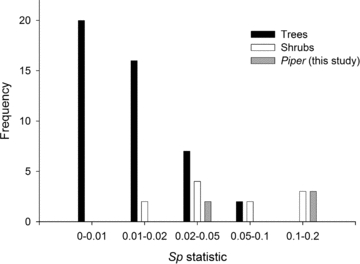
Histogram of the distribution of “sp” values reported for 11 temperate species and 34 tropical tree species and for one temperate and five tropical shrub species, and for the five *Piper* species studied here.

This pattern of strong SGS within populations is also reflected in the *Sp* statistic (Table 2). The greatest structure existed when ramets (including clones) were analyzed in *P. darienensis* and *P. cordulatum*, two of the understory species with high asexual recruitment, where *Sp* ranged from 0.075 to 0.152; and in *P. marginatum*, one gap species where *Sp* = 0.191. The least structure was observed for genets only, where most values of *Sp* ranged from 0.01 to 0.04, with the exception of *P. marginatum* and *P. cordulatum*, which showed the greatest structure at the level of the genet analysis (*Sp* = 0.131 and *Sp* = 0.100, respectively), probably as a result of its populations having aggregations dominated by related neighboring plants. These values of *Sp* for *Piper* are higher than those reported for 45 species of trees and six species of shrubs (Fig. 2).

### Population structure and gene flow

Analyses of population structure using *Fst* and Ф_pt_ indicated strong genetic differentiation in the five *Piper* species over relatively short geographic distances; 750 m for understory species and 200 m for light-demanding species (Table 3). Overall, indices of population subdivision (*Fst*) were significant and were in the range of 0.11–0.46. Values of Ф_pt_ diverged significantly from zero, based on the permutation test and were in the range of 0.14–0.49 (Table 3).

**Table 3:** Summary table of *Ф_pt_* and *Fst* values for all species and plots

Pairwise comparisons	Distance between populations	*Ф_pt_*	*Fst*		
			
Inbreeding coefficient			*F_is_* 0	*F_is_* 0.5	*F_is_* 1
*P. darienensis*	750 m	0.28^*^^*^	0.22^*^^*^	0.27^*^^*^	0.27^*^^*^
*P. cordulatum*	750 m	0.14^*^^*^	0.11^*^^*^	0.13^*^^*^	0.13^*^^*^
*P. aequale*	750 m	0.49^*^^*^	0.36^*^^*^	0.44^*^^*^	0.46^*^^*^
*P. marginatum*	200 m	0.23^*^^*^	0.18^*^^*^	0.22^*^^*^	0.22^*^^*^
*P. dilatatum*	200 m	0.18^*^	0.12^*^^*^	0.13^*^^*^	0.14^*^^*^

Estimates of *Fst* were calculated assuming three inbreeding coefficients (*F_is_* = 0 for random mating to *F_is_* = 1 for complete selfing).

Populations were found to be more strongly genetically differentiated than expected at random (^*^P < 0.01; ^*^^*^P < 0.0001).

**Figure 3:**
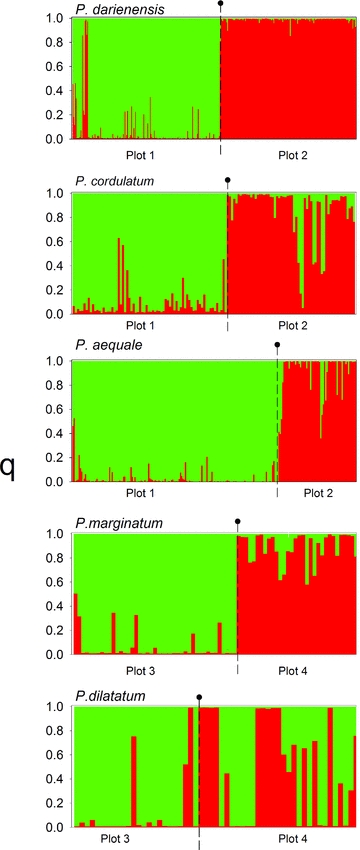
Estimated population structure using the Bayesian algorithm in the software *Structure* (ver. 2.2). Each individual is represented by a thin vertical bar, which is partitioned in red and green segments in proportion to the estimated ancestry in each source plot. Green for Plots 1 and 3 and red for plots 2 and 4. The dotted line indicates the separation between plots.

Likewise, from the *Structure* analysis, we found two clearly distinct populations (*K* = 2) for all species with little admixture for four of the five species studied (Fig. 3). *Piper dilatatum* was the species with more genetic admixture, many of their individuals showed mixed ancestry with a high proportion of individuals genomes (q) derived from the other population (Fig. 3).

**Figure 4:**
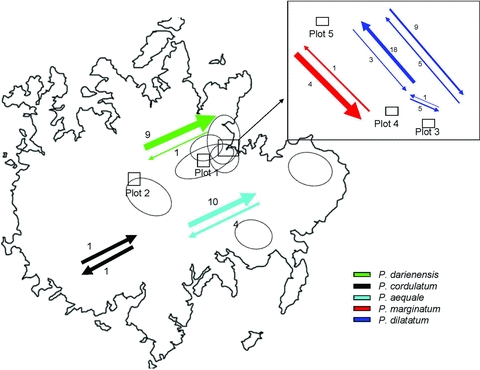
Map of Barro Colorado Island showing the location of the plots in the understory (plot 1 and plot 2) and in the lab clearings (plot 3 and plot 4). Plots 1 and 2 were 754 m apart, and Plots 3 and 4 were 200 m apart. Arrows show the direction of gene flow and the adjacent numbers are the number of immigrants or individuals with recent immigrant ancestors (with significant high probabilities) moving between populations. Dotted circles represent the foraging area of several radio-tracked *Carollia castanea* bats from Thies (1998).

Patterns of gene movement were similar for the understory species. For *P. darienensis*, 10 of 349 individuals (2.9%) were found to be immigrants or to have had an immigrant ancestor. Most of them (9/10) were immigrants coming from plot 2 to plot 1 (Fig. 4). Similarly for *P. aequale*, 14 of 225 individuals (6.2%) were immigrants or descendents of immigrants, with most migration (10/14) from plot 2 to 1. For *P. cordulatum*, only two of 132 individuals (1.5%) were immigrants or descendents of immigrants (Fig. 4). The patterns of gene movement were different for the two light-demanding species. For *P. marginatum*, five of 84 individuals (5.9%) were found to be immigrants or had an immigrant ancestor. Most (4/5) moved from plot 4 to plot 3. On the other hand, *P. dilatatum* had the largest number of immigrants; 21 of 76 (27%). For this species, the majority of movement was in the opposite direction of the movement observed for *P. marginatum* (Fig. 4).

## Discussion

### Spatial genetic structure

All five species of shrubs in the genus *Piper* examined here showed strong SGS, at both fine- and large-spatial scales. At the fine scale, we found *Sp* values, a measure of the strength of SGS, that are higher than those reported for all tropical trees studied so far (Fig. 2; Dutech et al. 2002; Vekeman and Hardy 2004; Hardy et al. 2005; Jones and Hubbell 2006; Cloutier et al. 2007; Dick et al. 2008; Bizoux et al. 2009). Ideally, we would have also estimated gene dispersal distance, σ^2^, and compared this value with those reported for trees. However the measures of effective population density needed to calculate σ^2^ are not available for our populations, and estimates from measured density are not reliable (Hardy et al. 2005). None the less, *Sp* has proved to be a good synthetic statistic for comparative purposes (Vekemans and Hardy 2004), and thus the striking differences in *Sp* between the majority of trees and the few shrubs studied so far suggests fundamental differences between these two groups. High *Sp* values can arise when either dispersal is strongly constrained or when the effective density is low. We discard the second option because *Piper* populations occur at higher densities (∼70–186 individuals per ha) than the vast majority of trees (0.12 to 21 individuals per ha; Hardy et al. 2005) and yet our *Sp* values are greater than those reported in those trees.

At a larger-scale, our values of *Fst*, ranging between 0.10 and 0.46 are also in marked contrast to those obtained for other tropical species. These typically range from 0.034 to 0.17 for populations separated by hundreds of meters to kilometers (Hamrick and Loveless 1986, Hamrick et al. 1993, Loiselle et al. 1995; Dutech et al. 2002; Dick et al. 2008). The low *Fst* in these published studies has been interpreted as evidence of extended gene flow in tropical tree populations. In contrast, our results, and those from earlier work with *Piper* species in Costa Rica and Brazil, show that strong genetic structure is a common characteristic of *Piper* species (Heywood and Fleming 1986; Mariot et al. 2002) and probably results from the combined effects of limited seed dispersal (Fleming 1981; Fleming and Heithaus 1981), limited pollen dispersal (Kikuchi et al. 2007), and the frequency of selfing (Figueiredo and Sazima 2000). Earlier studies in *Piper*, however, have not evaluated the contribution of clones to SGS; here, we show that clonality also significantly increases SGS.

After excluding clones SGS still remains significant and pairwise kinship values remain high, indicating that nonclonal neighboring plants are also genetically related. Values of pairwise kinship above 0.125 are expected for individuals that are half-siblings. Our results indicate that neighboring plants originated either from seeds of the same plant (limited seed dispersal), from seeds sired by self-pollen or pollen of relatives (limited pollen flow), or from both. The observation that *P. marginatum*, a self-incompatible species, showed similar SGS to the other self-incompatible species suggest that self-fertilization alone is not the cause of the high levels of population differentiation observed.

Although limited gene flow is responsible for part of the strong SGS observed, recruitment of clones increases the magnitude of spatial autocorrelation in *Piper* populations as it does in other clonal plant species (Chung and Epperson 1999; Chung et al. 2005; Jacquemyn et al. 2005) and in clonal sessile marine animals (Calderon et al. 2007). It is clear that clonal spread contributes to strengthening spatial genetic structure and any conclusion about limited gene flow would be misleading if identification of clones and its effects on the genetic structure were not considered. Likewise, disregarding limited gene flow as an important source of genetic structure in clonal organisms could result in misleading ecological and evolutionary interpretations.

### Integration of behavioral and molecular approaches to understanding gene flow

Models of gene flow have been based on a variety of approaches: behavioral observations of pollinators and seed dispersers (Webb and Bawa 1983; Boshier et al. 1995; Russo et al. 2006), indirect estimates of genetic differentiation obtained from molecular data (e.g., Hamrick et al. 1993; Dick et al. 2003; Vekemans and Hardy 2004), DNA fingerprint matching of maternal tissue attached to dispersed seeds to that of mapped trees (e.g., Jones et al. 2005; Jones and Muller–Landau 2008), and measuring pollen flow through parentage and TWOGENER analysis (Smouse and Sork 2004; Dick et al. 2008). Use of a combination of approaches is desirable since indirect methods or behavioral observations are not always good predictors of gene dispersal (Willson 1993; Hardesty et al. 2006). Here, we compare our genetic estimates of population differentiation with (1) radio-tracking data on the movement patterns of *Carollia castanea*, the principal disperser of understory *Piper* seeds, obtained in the same area where our plots were located (Fig. 4; Thies 1998) and (2) estimates of pollen dispersal obtained by tracking the movement of fluorescent powder on the *P. dilatatum* population studied here (Kikuchi et al. 2007). Both sets of data are in agreement with our genetic estimates and point to restricted gene flow in *Piper*.

The main dispersers of *Piper* seeds in most parts of the Neotropics are *Carollia perspicillata* and *Carollia castanea* (reviewed by Thies 1998), although *Piper* species that occur in gaps can also be dispersed by birds and ants (Thies and Kalko 2004). Here, we summarize published literature on the movement patterns of *Carollia castanea* on BCI (Thies 1998), and *Carollia perspicillata* on BCI and Costa Rica (Fleming 1988; Meyer 2007). Both *Carollia* species carry fruits away from the parent plant and process them at temporary night-roosts where hundreds to thousands of tiny *Piper* seeds are excreted intact (Fleming 1988; Thies 1998), probably leading to a clumped dispersal distribution of related seeds, corresponding to the fine-scale genetic signal we observe.

*Carollia* behavior is also consistent with the larger-scale genetic structure we observe in *Piper* populations. *Carollia castanea* foraging areas range from 5 to 100 ha, and are used repeatedly night after night. However, they spent 70–90% of the time in core areas of only 0.5–12 ha (Thies 1998). The restricted movement of bats within their home range, and the regular use of the same flight ways may explain the marked population structure found in this study. If the populations we sampled are inside the home ranges of different bats, and their ranges do not overlap, then the different genetic composition of the two sampled plots may reflect the genetic composition of sets of plants that are regularly visited by different groups of bats. In Figure 4, we illustrate the location of the sample plots relative to the foraging areas of 13 individuals of *C. castanea* obtained by Thies (1998) on BCI. This indicates that the distance between our understory plots is larger than the diameter of the foraging area of most bats studied. Using radio tracking data and gut passage time of swallowed seeds, Thies (1998) also estimated the probability distribution of seed dispersal distances and found that the highest probability of seed deposition is 100–220 m from the fruit source and declines to very low levels for distances >800m—the distance separating our two plots. Thus, molecular and behavioral data suggest that *Piper* seeds are dispersed in clumps, and that clumps are most likely within a few hundred meters of the maternal plant. Although we lack detailed data on the home range and foraging area of *C. perspicillata*, capture–recapture data over a 2-year period from Meyer (2007) show that the species also have a restricted movement, moving on average 0.7 km

Despite the apparent low mobility of *Carollia* (Meyer 2007), some long distance dispersal events seem to take place as shown by the *Structure* analysis (Fig. 3), where we identified a few across-plot migrants. These migrants may result from exploratory flights of bats in search of sparsely distributed resources (Westcott and Graham 2000) at the end of the fruiting period when resources are limited. How many of these long distance movements are necessary to prevent population differentiation is unknown, but given the consistency and predictability of *Piper* fruit production, which is spread across many small individuals (Thies 1998), we suspect they are likely to be infrequent.

In clearings around the lab, given the shorter distances among sample plots and their location within the foraging areas of many different bats (Thies 1998, Fig. 4), we expected to see high gene movement and low population differentiation. Observations for *P. dilatatum* were closer to this expectation, but, surprisingly, for *P. marginatum*, we still found high genetic differentiation between plots located less than 200 m apart. These clearings have been maintained as open areas for several decades, and the populations established in them might be rather old, at least in comparison to those in forest gaps. Strong initial genetic structure in recent gaps may also arise as a consequence of a founder effect that is further enforced by restricted gene flow in *Piper* species with low recruitment success (Greig 1993; Lasso et al. 2009). Fruits of *Piper* gap species can also be removed by birds (3.8% of fruits), and ants (26%) (Thies and Kalko 2004) both of which usually move seeds very short distances (1—12 m; Heithaus 1986; Passos and Ferreira 1996; Leal and Oliveira 1998). Furthermore, the birds visiting *Piper*, particularly tanagers, tend to mash the fruit, and drop the seeds near parent plant. We found that in both environments, understory and clearings, plants seem to be surrounded by relatives, suggesting that in all five species clumped dispersal by bats, and short-distance dispersal by birds and ants is taking place. In all populations, continued survival will depend in large degree on their ability to mate with relatives.

The most common visitors to *Piper* flowers consist of Hymenoptera and Diptera, particularly bees of the genus *Megachile* and *Trigona*, and Syrphid flies (Semple 1974, Fleming 1985, Figueiredo and Sazima 2000; Kikuchi et al. 2007). Observations of pollinator behavior and pollen tracking using fluorescent powder indicates that pollinators move pollen mostly up and down inflorescences and only occasionally between inflorescences and neighboring plants (Semple 1974; Kikuchi et al. 2007). This contrasts with genetic analyses that indicate that small insects can transport pollen very large distances (>10 km) between individual tropical trees and populations (Nason et al. 1998; Dick et al. 2008). These estimates, however, are for trees that generally occur in low densities (Hubbell and Foster 1983), favoring longer pollinator movements (Stacey et al. 1996), and which tend to be self-incompatible (Bawa 1979; Bawa et al. 1985). Whether the high density aggregation of shrubs leads to different pollen movement patterns from those of trees needs to be determined from offspring arrays and paternity analysis.

### Ecological and evolutionary implications

The establishment of genetically related near-neighbors and identical ramets has implications for demographic and reproductive processes. Aggregations of closely related individuals may facilitate the transmission of diseases (Burdon and Chilvers 1982). Likewise, the likelihood of inbreeding will increase if neighboring flowering plants are related or are clonemates, as was found here. Because pollen tends to move shorter distances when flowering plants are clumped (Stacey et al. 1996; Vekemans and Hardy 2004), mating among related near-neighbors could be common, leading to widespread self-fertilization, and raising the potential for inbreeding depression (Hufford and Hamrick 2003). This could explain the low seed viability found for some of the understory *Piper* species (Greig 1993; Lasso et al. 2009), which are mostly self-compatible species (Kikuchi et al. 2007; E. Lasso unpublished data).

From our population genetic data, we know that little gene flow occurs between sampling plots 750 m apart. From radio-tracking data, we know that most seeds of *Piper* should only disperse around 100–200 m (Thies 1998), from fluorescent dye studies we know that pollen movement in *Piper* is mostly restricted to within 50 m (Kikuchi et al. 2007), and by identifying clones with molecular markers we know that dispersal by asexual reproduction is mostly limited to within 5–10 m (Lasso et al. 2011). All these mechanisms of gene dispersal in *Piper* seem to lead to restricted gene flow, which could potentially allow diversification of populations in response to adaptation or genetic drift. Although population differentiation does not automatically lead to reproductive isolation, which is a prerequisite for speciation, it is still possible that part of the mechanism behind the high species diversity of this large shrub genera is related to founder effects (Mayr 1963) followed by restricted gene flow, drift, and/or natural selection as Baker (1959) and Fedorov (1966) hypothesized long ago.

Results presented here do not provide a definitive test of the BF hypothesis; that would require data from a larger array of tropical shrub species and sites. None the less, our study highlights the fact that the BF hypothesis remains inadequately tested, and that assumptions about the breeding systems and spatial genetic structure of tropical plant populations based on data for canopy trees cannot be uncritically extended to shrubs. Our dataset supports one key prediction of BF by showing a consistent pattern of strong genetic structure across five congeneric species. Whether we can generalize from our data to other species or populations remains open because our samples come from only a few populations that are located on an island and in relatively young forest. None the less, studies of the frugivorous bat community around BCI have shown that islands harbors similar species and abundances to those on the mainland (Meyer and Kalko 2008). It is also possible that the history of land use play a role in the results observed, as the forest sites we selected were disturbed in the 1800s (Foster et al. 1996). However, the fact that strong structure remains several generations after forest disturbance suggests that population mixing is very limited and founder effect is not the only explanation of the genetic structure observed.

### Determinants of diversification in trees versus shrubs

Ecologists have long recognized that there is no single ultimate mechanism that can account for the latitudinal richness gradient; instead they suggest that several contributory mechanisms may be in action (Gaston and Blackburn 2000, Willig et al. 2003). Some combination of existing hypotheses likely explains the high diversity of both trees and shrubs in the tropics. However, distinct mechanisms might have primacy for each species group. Trees and shrubs may be subjected to different ecological and evolutionary processes that are worth evaluating, and that may be linked to the observation that hyper-diverse genera (>700 species) of tropical plants are overwhelmingly composed of shrubs (*Psychotria*, *Piper*, *Miconia*), or understory herbaceous taxa (e.g., *Philodendron*, *Peperomia*). Likewise, families that include both shrubs and trees have much higher diversity in shrub than canopy genera (e.g., the two highest diversity palm genera in the neotropics are understory shrubs *Geonoma* (64 species) and *Chamaedorea* (107 species).

Trees differ from shrubs in many characteristics (population densities, breeding system and compatibility systems, and body size of seed dispersers) that may lead to greater seed and pollen movement in trees relative to shrubs, which in turn should slow population differentiation and, possibly, sympatric speciation. Moreover, differences in intrageneric diversity between trees and shrubs may reflect different speciation rates among groups. More evidence has recently emerged that suggests that rates of mutation and molecular evolution changes with life history in flowering plants (Smith and Donoghue 2008), between woody and herbaceous species (Kay et al. 2006) and between annuals and perennials (Andreasen and Baldwin 2001). Although the underlying mechanisms are unclear, shorter generation times (as occurs in shrubs relative to trees) seems to be associated with a faster rate of molecular evolution (Ohta 1993; Kay et al. 2006). Moreover, in shrubs that reproduce clonally, evolutionary rates may be faster because somatic mutations can be passed asexually to “offspring” from nonreproductive individuals. The lower diversity of temperate shrub genera, may then be attributed to a lower rate of molecular evolution (Wright et al. 2006) and speciation (Mittelbach et al. 2007). Higher ambient temperatures, higher mutation rates, shorter generation time, faster physiological processes (Rohde 1992; Allen et al. 2006), and diversity itself (Emerson and Kolm (2005) seem to be promoting more speciation in tropical shrubs in comparison to temperate shrubs.

The tropical rain forest contains strikingly large numbers of plant species, many of which are closely related sympatric species and many might be accounted for by explosive speciation (Gentry 1989). Hyperdiverse genera composed mostly by shrubs or small trees such as *Eugenia* (∼1,000 species), *Miconia* (>1,000 species), *Piper* (>1,000 species), *Inga* (∼300 species), and *Psychotria* (∼2,000 species) contribute disproportionally to total diversity in the tropics. Many congeners in these groups also occur sympatrically and appear to have similar morphology, reproductive syndromes, and habitat preferences, raising yet again the enigmatic issue of coexistence and speciation (Kursar et al. 2009). Some of these species-rich genera, such as *Inga* diversified recently (2–10 Ma; Richardson et al. 2001), while others like *Piper* and *Psychotria* are relatively old and go back into the Eocene (∼40 Ma; Wikström et al. 2001; Paul et al. 2009), or even farther; the latest *Piper* fossil found comes from the cretaceous (∼70 Ma; Martinez C. unpublished). For these old genera, part of their tremendous species diversity can probably be ascribed to their age and pantropical distribution, yet the existence of many *Piper* and *Psychotria* sympatric species of small geographic range and relatively recent origin (3–12 Ma; Paul and Tonsor 2008; Paul et al. 2009) suggests that other processes have driven rapid speciation in these large genera. Hypotheses to account for this, include adaptation to differences in the abiotic environment, pollinators or seed dispersers, or interactions with herbivores, as seems to be the case for *Inga* (Kursar et al. 2009). The pattern of genetic structure observed here could be also consistent with these hypotheses if populations are subjected to different adaptive pressures and different set of genotypes succeed in each population.

Here, we propose yet another mechanism that that may contribute to diversification in *Piper*, and perhaps to other species-rich genera. This mechanism represents a modified version of BFH that adds extended clonal growth to the limited gene flow and genetic drift proposed by them to favor the establishment and subsequent survival of small reproductively isolated populations—the conditions under which much of the speciation in tropical woody plants has probably occurred (Leigh et al. 2004). Members of other species-rich genera have shown some ability to resprout from fragments (Kinsman 1990; Bellingham et al. 1994) and, like *Piper*, are commonly found in aggregations and are also animal-dispersed. Some, however, like *Inga* and *Psychotria* differ from *Piper* in their mating systems and include many species that are self-incompatible (Koptur 1984) or exhibit heterostyly (Sakai and Wright 2008).

Whether restricted gene flow also occurs in those speciose tropical shrub genera remains an open question. Loiselle et al. (1995) studied one *Psychotria* species using allozymes, a marker less variable than AFLP, and found low but significant values of genetic differentiation among subpopulations separated by only 120 m, and detected that neighboring plants were either siblings or parent and offspring; again evidence of limited dispersal. Evidence from another six tropical shrubs species, together with these five *Piper* species indicates that shrubs have more restricted gene flow than the majority of trees studied so far (Fig. 2). Whether low dispersal increases opportunities for speciation through genetic isolation remains to be tested. Likewise, other fundamental questions arise and remain open; are shrub populations more likely to develop reproductive isolation than trees? Are there any indications that traits important in establishing reproductive isolation differ between shrubs and trees? Is somatic mutation more frequent in shrubs than in trees and does it spread faster in the population because of clonality? We hope that this paper stimulates a more thorough evaluation of the possibility that gene flow and other determinants of diversity differ between shrubs and trees allowing an assessment of whether the BFH should be revived as an explanation for the high species diversity of tropical shrubs.
